# Prioritizing Measures That Matter Within a Person-Centered Oncology Learning Health System

**DOI:** 10.1093/jncics/pkac037

**Published:** 2022-05-06

**Authors:** Aricca D Van Citters, Alice M Kennedy, Kathryn B Kirkland, Konstantin H Dragnev, Steven D Leach, Madge E Buus-Frank, Elissa F Malcolm, Megan M Holthoff, Anne B Holmes, Eugene C Nelson, Susan A Reeves, Anna N A Tosteson, the Promise Partnership Delphi Panel, Albert Mulley, Amber Barnato, Amelia Cullinan, Andrew Williams, Ann Bradley, Anna Tosteson, Anne Holmes, Anne Ireland, Brant Oliver, Brock Christensen, Carol Majewski, Carolyn Kerrigan, Catherine Reed, Cathy Morrow, Corey Siegel, Daniel Jantzen, David Finley, Elissa Malcolm, Elizabeth Bengtson, Elizabeth McGrath, Elizabeth Stedina, Ellen Flaherty, Elliott Fisher, Eric Henderson, Erick Lansigan, Evan Benjamin, Gabriel Brooks, Garret Wasp, George Blike, Ira Byock, Janet Haines, Jenn Alford-Teaster, Jenna Schiffelbein, Jennifer Snide, Joanna Leyenaar, Jocelyn Chertoff, Joga Ivatury, Johanna Beliveau, John Sweetenham, Judith Rees, Julia Dalphin, Julie Kim, Karen Clements, Kathryn Kirkland, Kenneth Meehan, Konstantin Dragnev, Kris Bowen, Lawrence Dacey, Linton Evans, Malavika Govindan, Marcus Thygeson, Martha Goodrich, Mary Chamberlin, MaryAnn Stump, Matthew Mackwood, Matthew Wilson, Meredith Sorensen, Michael Calderwood, Paul Barr, Polly Campion, Ralph Jean-Mary, Rian M Hasson, Sai Cherala, Sally Kraft, Samuel Casella, Samuel Shields, Sandra Wong, Shoshana Hort, Stephanie Tomlin, Stephen Liu, Stephen LeBlanc, Steven Leach, Susan DiStasio, Susan Reeves, Virginia Reed, Wendy Wells, Whitney Hammond, Yolanda Sanchez

**Affiliations:** Geisel School of Medicine at Dartmouth, The Dartmouth Institute for Health Policy and Clinical Practice, Lebanon, NH, USA; Geisel School of Medicine at Dartmouth, The Dartmouth Institute for Health Policy and Clinical Practice, Lebanon, NH, USA; School of Health and Welfare, Jönköping University, Jönköping, Sweden; Geisel School of Medicine at Dartmouth, The Dartmouth Institute for Health Policy and Clinical Practice, Lebanon, NH, USA; Section of Palliative Medicine, Department of Medicine, Dartmouth Health, Lebanon, NH, USA; Department of Medicine, Geisel School of Medicine at Dartmouth, Hanover, NH, USA; Department of Medicine, Geisel School of Medicine at Dartmouth, Hanover, NH, USA; Dartmouth Cancer Center, Dartmouth Health, Lebanon, NH, USA; Department of Medicine, Geisel School of Medicine at Dartmouth, Hanover, NH, USA; Dartmouth Cancer Center, Dartmouth Health, Lebanon, NH, USA; Department of Molecular and Systems Biology, Geisel School of Medicine at Dartmouth, Hanover, NH, USA; Geisel School of Medicine at Dartmouth, The Dartmouth Institute for Health Policy and Clinical Practice, Lebanon, NH, USA; Section of Neonatology, Department of Pediatrics, Dartmouth Health, Lebanon, NH, USA; Analytics Institute, Dartmouth Health, Lebanon, NH, USA; Geisel School of Medicine at Dartmouth, The Dartmouth Institute for Health Policy and Clinical Practice, Lebanon, NH, USA; Patient and Family Advisors, Dartmouth Health, Lebanon, NH, USA; Geisel School of Medicine at Dartmouth, The Dartmouth Institute for Health Policy and Clinical Practice, Lebanon, NH, USA; Department of Community and Family Medicine, Geisel School of Medicine at Dartmouth, Hanover, NH, USA; Dartmouth Health, Lebanon, NH, USA; Geisel School of Medicine at Dartmouth, The Dartmouth Institute for Health Policy and Clinical Practice, Lebanon, NH, USA; Dartmouth Cancer Center, Dartmouth Health, Lebanon, NH, USA

## Abstract

**Background:**

Despite progress in developing learning health systems (LHS) and associated metrics of success, a gap remains in identifying measures to guide the implementation and assessment of the impact of an oncology LHS. Our aim was to identify a balanced set of measures to guide a person-centered oncology LHS.

**Methods:**

A modified Delphi process and clinical value compass framework were used to prioritize measures for tracking LHS performance. A multidisciplinary group of 77 stakeholders, including people with cancer and family members, participated in 3 rounds of online voting followed by 50-minute discussions. Participants rated metrics on perceived importance to the LHS and discussed priorities.

**Results:**

Voting was completed by 94% of participants and prioritized 22 measures within 8 domains. Patient and caregiver factors included clinical health (Eastern Cooperative Oncology Group Performance Status, survival by cancer type and stage), functional health and quality of life (Patient Reported Outcomes Measurement Information System [PROMIS] Global-10, Distress Thermometer, Modified Caregiver Strain Index), experience of care (advance care planning, collaboRATE, PROMIS Self-Efficacy Scale, access to care, experience of care, end-of-life quality measures), and cost and resource use (avoidance and delay in accessing care and medications, financial hardship, total cost of care). Contextual factors included team well-being (Well-being Index; voluntary staff turnover); learning culture (Improvement Readiness, compliance with Commission on Cancer quality of care measures); scholarly engagement and productivity (institutional commitment and support for research, academic productivity index); and diversity, equity, inclusion, and belonging (screening and follow-up for social determinants of health, inclusivity of staff and patients).

**Conclusions:**

The person-centered LHS value compass provides a balanced set of measures that oncology practices can use to monitor and evaluate improvement across multiple domains.

For more than a decade, learning health systems (LHS) have been promoted as a means to improve health-care quality and health outcomes and narrow the divide between research and practice (1). LHS can optimize care processes, facilitate patient and clinician engagement in research, evaluate comprehensive data from existing sources, use data to support real-time decision making, and embed clinical trials into care delivery (2-7).

Rapid growth in oncology LHS has been supported by advances in the ability to harness data from multiple sources to quickly understand the impact of interventions on health and well-being of people with cancer, identify opportunities for improvement in care processes, and support public reporting (8-14). Despite this growth and the recognized need for rigorous measurement of LHS impact (15,16), a gap remains in identifying specific measures that can evaluate the longitudinal impact of an oncology LHS within a health-care system.

Although numerous performance indicators exist, measurement sets often narrowly focus on impact of treatment on clinical outcomes (14), subpopulations of people living with cancer (17), or single domains of quality (18,19). The onus has fallen on individual systems to identify key indicators of an effective LHS based on their focus of interest.

The clinical value compass (20) offers a framework for measuring a balanced set of critical process and outcome indicators that have the greatest importance to multiple stakeholders in evaluating health-care quality and value. The objective of our study was to use a multistakeholder process to define key domains and prioritize measures within each domain to create a clinical value compass for an emerging person-centered oncology LHS.

## Methods

We conducted a 3-round modified Delphi process (21,22) to identify a balanced measure set to evaluate the progress and impact of a person-centered oncology LHS. The modified Delphi process was conducted between July and September 2020, with oversight by a 19-member Data, Measurement, and Scholarship Workgroup representing diverse stakeholders within the health system (researchers, people living with cancer, clinicians, leaders).

### Setting

This work occurred in a National Cancer Institute–designated comprehensive cancer center in northern New England, serving a largely rural catchment area. The cancer center treats approximately 32 000 patients annually and includes 17 interdisciplinary clinical oncology groups serving discrete populations.

At the time of this undertaking, the cancer center and its larger health system and affiliated health services research organization were preparing to launch The Promise Partnership Oncology LHS to advance the health system’s strategic plan. The Promise Partnership LHS has 3 primary aims: promoting continuous improvement of health service quality, advancing cancer research, and supporting cancer care with and for people living with cancer. It is designed to optimize care processes by fostering mutually beneficial partnerships between people living with cancer, their families and support networks, and health professionals. Initial projects are designed to enhance care experiences and joy and fulfillment in work among health professionals.

### Recruitment

We used purposive sampling to identify a multidisciplinary group of stakeholders to participate in the modified Delphi process with the intent of achieving input from 6 stakeholder groups (Table 1). Potential participants were selected from the health system, cancer center, health services research organization, people living with cancer and family members, and external partners. No participation incentives were provided.

**Table 1.** Overview of Delphi process participation by multidisciplinary group of stakeholders

Stakeholder group	Ballot 1		Ballot 2		Ballot 3	
	Ballot completion	Discussion attendance	Ballot completion	Discussion attendance	Ballot completion	Discussion attendance
	No. (%)	No. (%)	No. (%)	No. (%)	No. (%)	No. (%)
People living with cancer and family members (n = 7)	7 (100)	7 (100)	7 (100)	7 (100)	7 (100)	6 (85.7)
Clinicians and other clinical staff (n = 19)^a^	17 (89.5)	17 (89.5)	19 (100)	15 (78.9)	17 (89.5)	11 (57.9)
Institutional leaders (n = 19)^b^	18 (94.7)	14 (77.8)	17 (94.4)	11 (61.1)	19 (100)	9 (47.4)
Improvement leaders (n = 9)	8 (88.9)	4 (44.4)	9 (100)	7 (77.8)	9 (100)	7 (77.8)
Researchers (n = 16)^c^	16 (100)	13 (81.3)	15 (93.8)	15 (93.8)	16 (100)	13 (81.3)
Policy makers and funders (n = 7)	7 (100)	4 (57.1)	5 (71.4)	3 (42.9)	5 (71.4)	3 (42.9)
Total participants (n = 77)	73 (94.8)	59 (76.6)	72 (93.5)	58 (75.3)	73 (94.8)	49 (63.6)

^a^ Clinicians and other clinical staff included the following subgroups: physicians (n = 12) and clinical staff (eg, nursing, social work, chaplaincy) (n = 7).

^b^ Institutional leaders included subgroups:  system or cancer center leaders (n = 10) and Clinical Oncology Group practice leaders (n = 9).

^c^ Researchers included subgroups: clinician researchers (n = 8) and nonclinician researchers (n = 8).

### Domain and Measure Identification

The initial measurement framework was informed by the condition-agnostic clinical value compass domains (20,23) and complementary measurement domains that reflect the LHS ecosystem (16,24). Seven domains were identified a priori: 1) clinical outcomes; 2) functional health outcomes; 3) experience of care; 4) cost, resource use, and financial indicators; 5) team well-being and joy in work; 6) learning culture and community; and 7) scholarly engagement and productivity. An eighth domain was added following round 1 of the Delphi process, reflecting diversity, equity, inclusion, and belonging.

Three rounds of blinded voting occurred in July, August, and September 2020 via online ballots (Qualtrics, www.qualtrics.com). First-round ballots included an extensive list of subdomains within each domain. Second- and third-round voting explored, then narrowed, candidate measures. The research team provided each participant with comprehensive supplementary materials to support voting decisions and was available by phone or e-mail to answer questions. Each round of voting was followed by a 50-minute virtual video meeting to discuss voting results, creating opportunities for participants to advocate for lower-ranked measures and contribute alternate measures that may have been overlooked. Discussions were recorded with participants’ knowledge and consent. We used descriptive statistics to analyze survey results using SPSS (version 26.0).

### Ballot Content

Ballot 1 (see the Supplementary Materials, available online) prioritized measurement subdomains within 7 LHS domains. Fifty-three subdomains were presented based on a prototype value compass for serious illness care (23), the quadruple aim (25), the Institute for Healthcare Improvement’s Whole System Measures (26), and a targeted review of measurement within each domain. Participants were asked to rank importance of subdomains on a 1-9 scale (1 = extremely unimportant; 9 = extremely important). To account for potential ceiling effects in Likert ratings, participants were asked to identify the 2 most important subdomains within each domain. Ballot 1 was distributed via email and available for 7 days. Median time of completion was 17 minutes.

Ballot 2 (see the Supplementary Materials, available online) identified and ranked measures within each subdomain that advanced from Ballot 1. Subdomains advanced to round 2 based on a combination of mean score, number of respondents categorizing the subdomain as a priority, and prioritization during facilitated discussion. The following sources were consulted to identify potentially relevant measures for each subdomain: National Quality Forum (27), Consumer Assessment of Health Plans Study surveys (28), National Committee for Quality Assurance (29), Lown Institute Hospitals Index (30), *US News and World Report* cancer-specific metrics (31), Commission on Cancer Accreditation Measures (32), Quality Oncology Practice Initiative (33), Institute for Healthcare Improvement Whole System Measures white paper (26), New Hampshire State Cancer Registry (34), and Measures of Person-Centered Coordinated Care (35). Measures were also identified through targeted literature reviews, input from the panel, and measures available from the cancer center or health system. Candidate measure inclusion criteria included being valid, reliable, sensitive to change, feasible to measure, and aligned with values and priorities of the LHS. In domains with limited valid measures, conceptual definitions of measurement areas were included. Respondents were asked to rank a revised list of subdomains according to importance, then rate measures using a 1-5 Likert scale (1 = not at all important; 5 = extremely important). Ballot 2 was distributed via email and available for 7 days. Median time of completion was 48 minutes.

Ballot 3 (see the Supplementary Materials, available online) identified recommendations for the final set of measures. Measures that advanced to Ballot 3 had an average rating score greater than 4, were identified by more than 50% of Delphi panel members as a top priority, or were promoted during Delphi panel discussions. Ballot 3 asked respondents to rank measures within each domain. Ballot 3 was distributed via email and available for 6 days. Median time of completion was 21 minutes. An additional discussion session was held with Delphi participants living with cancer and family members to discuss Ballot 3 measures.

Recommendations for the final measurement set were derived from the proportion of respondents ranking a measure in the top 3 measures within a domain. The final discussion session focused on improving the selected measure set. Synthesis of discussion informed a set of draft recommendations presented to cancer center and health system leaders.

## Results

### Participants

Three-quarters (76%) of individuals invited to the measurement panel agreed to participate (77 of 103). Individuals represented 6 stakeholder groups: people living with cancer and family members, clinicians and clinical staff, health-care system or cancer center leaders, quality improvement leaders, clinician and nonclinician researchers, and policy makers or funders. Ballots were completed by 94% or more of participants in each round of voting (Table 1). Discussion group attendance ranged from 64% to 77%.

### Identification of Domains, Subdomains, and Measures

**Figure 1.**
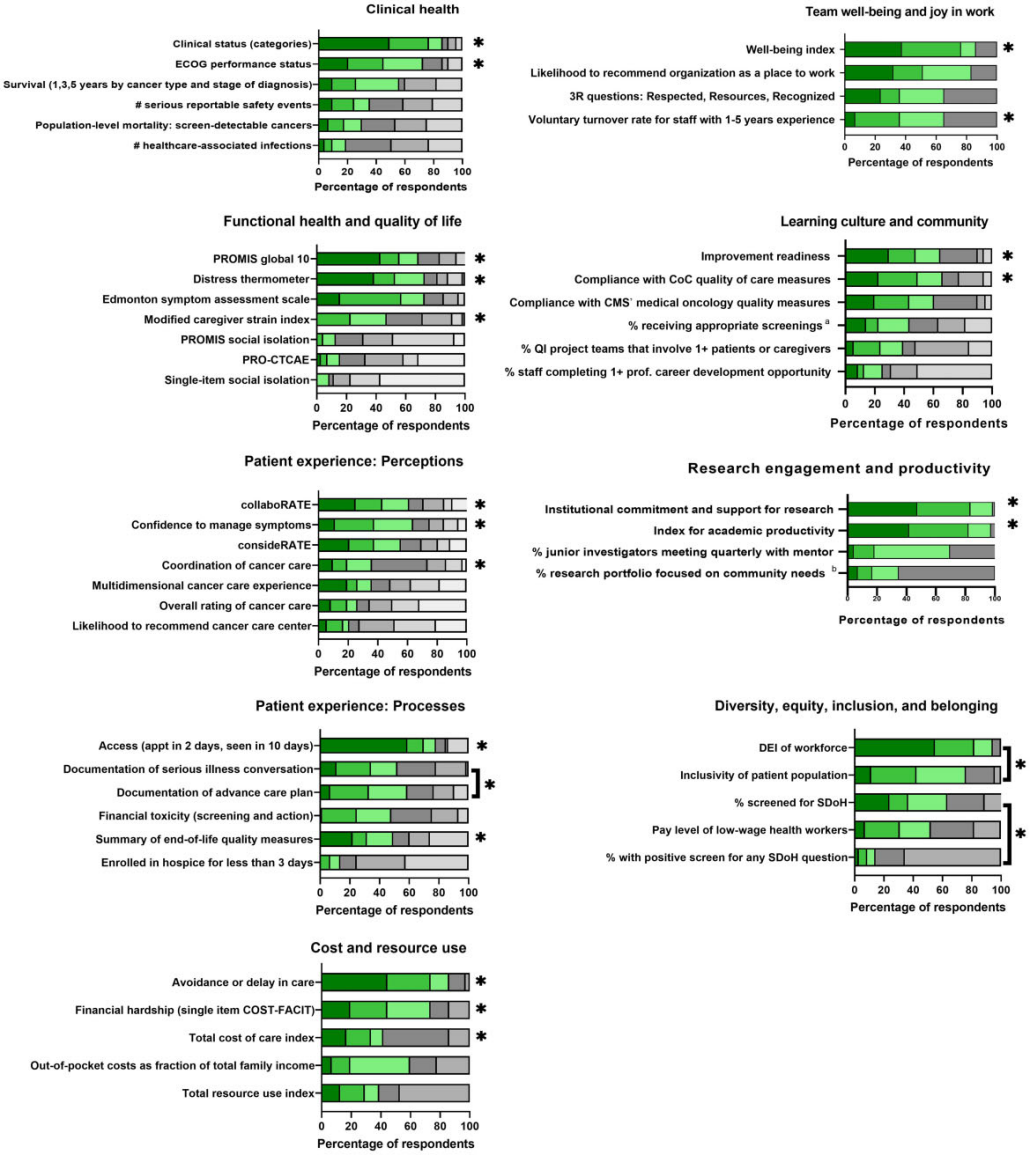
Prioritization of measures, according to ranking during round 3 voting. **Green shading** represents proportion of respondents ranking an item as first, second, or third most important within a domain. **Gray shading** represents proportion of respondents ranking an item as fourth through last within a domain. *Included within final recommendations. **Brackets** denote measures combined in final recommendation set. ^a^Screening for breast, cervical, and/or colorectal cancer and/or tobacco use and cessation interventions. ^b^Population health research includes health equity and disparity research, health promotion and disease prevention research, social determinants of health research, community health needs assessment, or community engaged research. CMS = Centers for Medicare & Medicaid Services; CoC = Commission on Cancer; COST-FACIT = COST-Functional Assessment of Chronic Illness Therapy; DEI = diversity, equity, and inclusion; ECOG = Eastern Cooperative Oncology Group; PRO-CTCAE = Patient-Reported Outcomes version of the Common Terminology Criteria for Adverse Events; PROMIS = Patient Reported Outcomes Measurement Information System; QI = quality improvement; SDoH: social determinants of health.

Round 1 voting and discussion (Supplementary Table 1, available online) resulted in 8 domains and 13 subdomains that drove selection of candidate measures for Ballot 2 (Box 1). In Round 2, 36 of 82 measures (44%) received a score of 4 or higher from two-thirds or more of participants (Supplementary Table 2, available online). Round 3 voting and discussion ranked the importance of 50 measures. Figure 1 shows prioritization of measures by domain (detailed ratings in Supplementary Table 3, available online).

Box 1. Domains and subdomains prioritized following ballot 1 voting and discussion

Health-related quality of life

- Patient-reported general well-being and quality of life, symptoms, or functional status
- Patient- or caregiver-reported burden, coping, and support

Clinical health outcomes

- Clinician-reported clinical improvement, disease activity, or symptoms and signs of illness
- Mortality, safety, or preventable harm

Costs, resource use, and health system financial indicators

- Quality measures of care at the end of life
- Costs of care
- Financial toxicity
- Financial health of organization

Experience of care

- Alignment of care with patient’s goals and preferences
- Access to care, continuity of care, and care integration
- Patient and family overall satisfaction with care

Team well-being and joy in work

Diversity, equity, inclusion, and belonging

Learning culture and community

- Using science and evidence to inform care decisions
- Culture of continuous improvement and innovation

Research engagement and productivity

Final Delphi panel recommendations for the value compass included 22 measures in 8 domains. Recommendations were presented to 1) the LHS data, measurement, and scholarship workgroup; and 2) senior leaders of the cancer center and health system for review and approval. These discussions resulted in 3 modifications: replacement of the claims-based total cost of care with the patient-focused economic analysis (due to availability of local data), inclusion of voluntary staff turnover (due to availability of historical data), and removal of the clinical improvement measure (due to lack of a field-defined variable within the electronic health record [EHR]).

**Figure 2.**
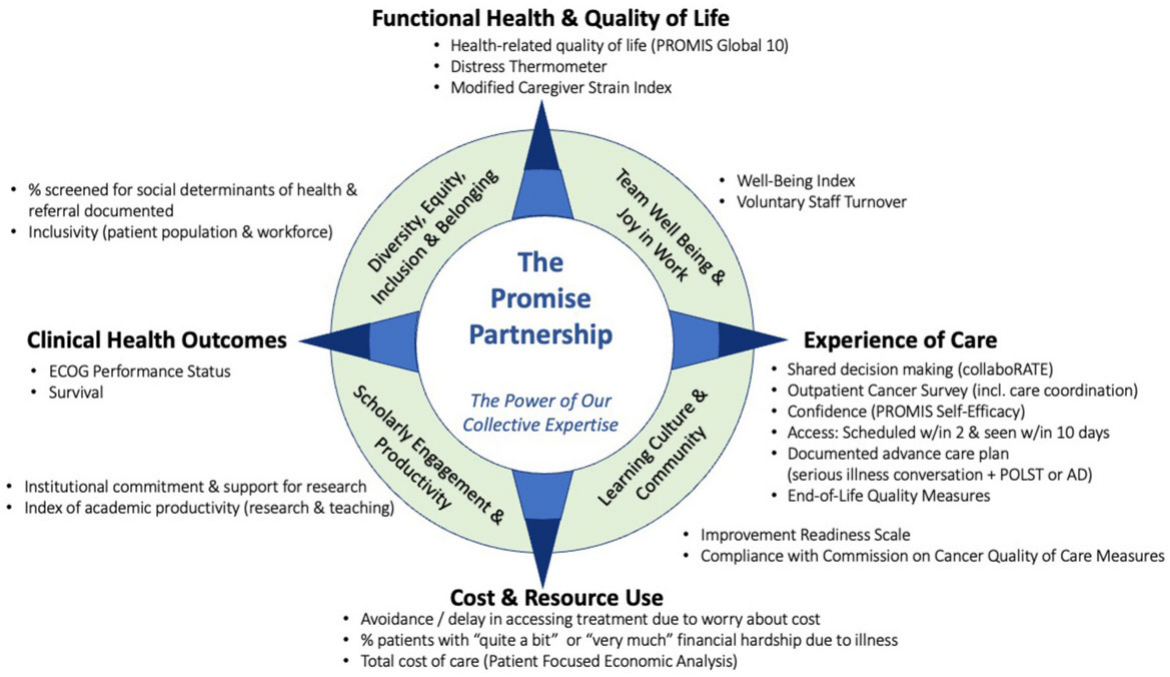
An oncology learning health system value compass. AD = advance directives; ECOG = Eastern Cooperative Oncology Group; POLST: physicians orders for life sustaining treatment; PROMIS = Patient Reported Outcomes Measurement Information System.

Figure 2 depicts a value compass comprising the final set of recommended measures to support the Promise Partnership Oncology LHS. Each domain is referenced by its “point” on the compass. Cardinal points (north, south, east, and west) represent factors associated with the patient and caregiver, and ordinal points (northeast, northwest, southeast, southwest) represent the newly emerging context and ecosystem of the LHS. Each domain includes 2 or 3 measures, except experience of care, which includes 6 measures (3 perception measures; 3 process of care measures). Measures are derived from clinical data within the EHR (n = 3), patient- or caregiver-reported data that can be embedded within the EHR or experience surveys (n = 9), administrative data (n = 6), clinician or staff data (n = 2), and registry or claims data (n = 2). Descriptions of measures, data sources, and reporting strategy are shown in Table 2.

**Table 2.** Description of final measure set, data sources, and reporting strategies^a^

Domain and measure	Description	Data source	Reporting strategy
Functional health and quality of life (north)			
PROMIS Global 10 (36)	Measure of health-related quality of life, including physical health, mental health, social health, pain, fatigue, and quality of life	Patient-reported data in EHR	Two composite scores: global mental health and global physical health
Distress Thermometer (37)	Screening tool for distress; allows clinician to tailor conversation around identified problems contributing to distress	Patient-reported data in EHR	Proportion of people with distress (score ≥4 on 0-10 scale), with referral to appropriate support or resources documented in EHR
Modified Caregiver Strain Index (38)	Screening tool of strain related to caregiving, including financial, physical, psychological, social, and personal strain domains	Caregiver-reported data	Total score (range = 0-26), where a higher score indicates higher caregiver strain
Experience of care (east)			
Documentation of ACP	Documentation of ACP discussion and process with patient and family, as illustrated by presence of serious illness conversation plus either advance directive or POLST noted within the EHR	Clinical data in EHR	Proportion of eligible people with specified documentation
collaboRATE (39)	Perception of shared decision making	Postvisit patient experience survey	Proportion of people providing top-box rating on all collaboRATE questions
PROMIS Self-Efficacy Scale for Managing Symptoms (40)	Confidence to manage symptoms during daily activities and in public places, to keep symptoms from interfering with relationships, and to work with clinician to manage symptoms	Patient-reported data in EHR	Overall composite score
Scheduled within 2 d of referral AND seen within 10 d of scheduling	New patients scheduled within 2 d of referral and seen within 10 d of scheduling at cancer center	Administrative data	Proportion of eligible people meeting specified criteria
Outpatient oncology survey (including coordination of care)	Measurement of experience of receiving cancer treatment, including categories for scheduling, registration, facility, radiation, chemotherapy, tests, oncologist, nurses, personal issues, and overall assessment	Postvisit patient experience survey	Proportion of people with top-box overall rating for care given at this facility
Summary score for end-of-life quality measures (27)	Measures of quality of care at end of life, including receipt of chemotherapy in last 14 d of life; proportion who died from cancer not enrolled in hospice; proportion who died from cancer enrolled in hospice for <3 d; proportion who died from cancer admitted to ICU in last 30 d of life; proportion with >1 emergency room visit in last 30 d of life; proportion with >1 hospitalization in last 30 d of life (NQF #0210–13; 0215–16)	Claims data	Proportion of people meeting criteria for all eligible end-of-life quality measures, based on 6 separate care use questions
Cost and resource use (south)			
Avoidance or delay in accessing care or medications due to worry about cost (43)	7 questions from CDC National Health Interview Survey Utilization and Prescription Medication core item banks assessing whether respondent delayed care in last 12 mo due to cost	Patient-reported data in EHR	Proportion or people responding yes to ≥1 of identified questions
Financial hardship (41,42)	1 item question assessing level of financial hardship: “My illness has been a financial hardship to my family and me”	Patient-reported data in EHR	Proportion of people responding “4 = quite a bit” or “5 = very much”
Total cost of care as measured by PFEA (44)	Total cost of care within health system as measured by PFEA, which is based on costing data calculated at billing code and/or encounter level (eg, CPT, DRG, etc)	Administrative data	Operating margin (comparison of cost of care with actual or estimated payments)
Clinical outcomes (west)			
ECOG performance status (45)	Describes patient’s level of functioning in terms of ability to care for self, daily activity, and physical ability (walking, working, etc)	Clinical data in EHR	Overall score, where a lower score is better
Survival (1, 3, and 5 y by cancer type and stage of diagnosis)	Length of time elapsed (y) between date of diagnosis or start of treatment for people living with cancer	Registry data	Proportion of people diagnosed or starting treatment 1, 3, and 5 y ago who are alive
Team well-being and joy in work (northeast)			
Well-Being Index (46)	Survey measures dimensions of employee burnout, fatigue, quality of life, depression, anxiety/stress, meaning in work, and time for personal/family life	Employee survey	Proportion of employees at risk for negative health consequences from distress
Voluntary turnover rate for staff	Measures when staff member willingly chooses to leave their position	Administrative data	Proportion of employees that voluntarily leave health system relative to average number of employees over the month
Diversity, equity, inclusion, and belonging (northwest)			
Percent of patients screened for social determinants of health, with follow-up documented in EHR	Measurement of degree to which system is assessing social determinants of health and providing follow-up referral or services to those that have identified issues	Patient-reported data in EHR	Proportion of people with a negative screen, or a positive screen and referral to appropriate support or resources documented in EHR
Inclusivity (patient and workforce)	Patient: extent to which health-care system’s patient population reflects demographics of community in which it is located, based on race, income, and education levels (using zip-code level data). Workforce: To be developed	Administrative data	Measurement under development
Learning culture and community (southeast)			
Improvement Readiness Scale (47)	Measurement of employee perception of learning environment’s readiness to support quality improvement	Employee survey	Proportion of employees reporting a positive (average >4) improvement readiness climate
Compliance with CoC Quality of Care Measures (32)	Performance on CoC quality of care indicators for bladder, breast, cervix, colon, endometrium, gastric, kidney, non-small cell lung, ovary, and rectum cancers	Clinical data in EHR	Proportion of eligible patients meeting all eligible quality-of-care indicators
Scholarly engagement and productivity (southwest)			
Institutional commitment and support for research	Index of amount of pilot funding through institutional mechanisms; mechanisms and money for protected research time; mentoring support of junior investigators; and investment in research education and support for clinical trials and related infrastructure, successful applications for extramural funding, and biostatistics and informatics support	Administrative data	Reporting strategy under development in alignment with existing measures
Academic productivity index (research and teaching)	Index of publications, grants, number of investigator-initiated clinical trials, community-based research projects, work that led to change in practice at our institution and beyond (eg, lung cancer screening program based on own team’s research findings), and time spent teaching others	Administrative data	Reporting strategy under development in alignment with existing measures

^a^ ACP = advance care plan; CDC: Centers for Disease Control and Prevention; CoC = Commission on Cancer; CPT = current procedural terminology; DRG = diagnosis related groups; ECOG = Eastern Cooperative Oncology Group; EHR = electronic health record; ICU = intensive care unit; NQF = National Quality Forum; PFEA = patient focused economic analysis; POLST = provider orders for life-sustaining treatment; PROMIS = Patient Reported Outcomes Measurement Information System.

Four domains and 14 measures are associated with the patient and caregiver. “Functional health and quality of life” includes measures reflecting overall well-being of people living with cancer and their caregivers. The Patient Reported Outcomes Measurement Information System (PROMIS) Global-10 (36) provides a global composite of health-related quality of life; Distress Thermometer (37) identifies distress among people with cancer and caregivers, and Modified Caregiver Strain Index (38) identifies impact of serious illness on caregivers. “Experience of care” includes shared decision making [collaboRATE (39)], coordination of care, and the PROMIS Self-efficacy Scale for Managing Symptoms (40) as well as access to care, documentation of advance care planning, and avoidance of aggressive end-of-life care (27). “Cost and resource use” assesses financial hardship because of illness (41,42), avoidance or delay in accessing treatment because of cost (43), and total cost of care measured through billing codes and encounter data (44). “Clinical outcomes” reflect clinician-rated functional status of the patient [ECOG Performance Status (45)] and population-level survival by cancer type and stage.

Four domains and 8 measures are associated with the LHS context and ecosystem. “Team well-being and joy in work” measures align with system-level data collection, including the Well-Being Index (46) and voluntary turnover rate among staff. “Diversity, equity, inclusion, and belonging” assesses the proportion of patients screened for social determinants of health with follow-up documented in the EHR and inclusivity among patients and workforce. “Learning culture and community” includes Improvement Readiness (47), which assesses ability of the work environment to support quality improvement, and a composite score of Commission on Cancer quality of care indicators (32). Finally, “scholarly engagement and productivity” assesses institutional commitment and support for research and academic productivity inclusive of research and teaching.

## Discussion

We identified a comprehensive and balanced set of 22 measures in 8 domains to guide the development and evaluation of a person-centered oncology LHS. The LHS value compass includes measures that matter to a diverse stakeholder group and is weighted toward experiences of care. This codesigned set of measures was developed to support evaluation, improvement, and scholarship within the developing person-centered oncology LHS, allowing data to be turned into information that answers important questions and can guide future actions. This measure set has relevance for studying care from multiple perspectives, including clinical care, health-care quality and value improvement, system performance monitoring and improvement, and population health. Measures complement and expand data regularly reported to leadership and those within CancerLinQ and state cancer registries (8–10,34).

The person-centered oncology LHS value compass facilitates a deliberate approach to measurement and reporting, feeding data back to multiple levels of the system (eg, clinical program level, cancer center system level, and health system level), and evaluating whether a balanced set of longitudinal measures can help drive practice-level and system-level improvement. The measure set includes leading and lagging indicators of performance for improvement interventions and the larger clinical enterprise. Leading indicators provide information on factors or processes important to achieving desired results (eg, Well-Being Index), and lagging indicators measure current performance (eg, voluntary staff turnover).

Our work aligns with principles for designing a learning measurement system, including scanning the landscape of existing measurement and implementation efforts, engaging key stakeholders from diverse sectors, and developing criteria for measure selection to guide care delivery, improvement, and science (48). Our prioritization of person-centered measures aligns with the coproduction LHS model (4,49) and recent work by others to develop indicators of effective person-centered oncology care (18,19). Our measure set improves on existing measure sets because of its codesigned origins, which resulted in a balanced set of measures associated with the patient and caregiver, alongside measures associated with the LHS ecosystem.

The traditional clinical value compass includes 4 domains: functional outcomes, patient experience, cost and resource use, and clinical outcomes. Functional outcomes offer opportunities to gauge the effectiveness of health interventions on patient and caregiver well-being and inform future interventions. Patient experience measures support our ability to align care with what matters most to people living with cancer and their caregivers. Cost and resource use measures emphasize financial toxicity to the patient and caregiver while enabling the health system to better understand value and variation in costs. Finally, clinical outcomes provide indicators of effectiveness of cancer care, with ECOG providing a means to stratify other analyses.

The codesign process generated a novel evolution of the clinical value compass, adding 4 priority areas for measurement. Team well-being and joy in work provides short- and long-term views of employee well-being. Diversity, equity, inclusion, and belonging measures support better understanding of factors that influence access, outcomes, and disparities in care and align with the evolving concept of the quintuple aim (50). Learning culture and community measures reflect our ability to support continuous improvement and innovation within the LHS while delivering guideline-driven care. Finally, scholarly engagement and productivity aim to measure constructs associated with the institution’s support of scholarship and associated scholarly activities.

This study has both strengths and limitations. Our Delphi panel comprised a large and diverse sample of stakeholders within the cancer community and beyond who participated actively in the work. Response rates to online ballots were consistently high (>94%), and discussion groups included an average of 72% of participants. Although we view this as a strength, the large size of the group presented some challenges for ensuring that all voices were heard. To address this, discussions were conducted with smaller subgroups, which allowed time for all individuals to speak. Because this multigroup discussion strategy limited the ability for panel members to participate in every discussion, we distributed recordings and transcripts of chats to all participants after each round of discussion. A strength and key feature of our Delphi process was inclusion of 7 people living with cancer or family members on the panel. To enhance engagement and psychological safety, we conducted a series of additional email interactions and a supplemental online video meeting with this group to invite and address questions and support candid conversations. We believe this led to more balanced participation in group discussions and to a stronger set of measures. Our panel included a higher proportion of individuals in clinical and research leadership roles and lower representation of staff nurses and other members of interdisciplinary teams that support cancer care delivery. This may have influenced measures selected and may affect uptake and use of measures. To support local acceptance and implementation of measures, membership in the Delphi panel was weighted toward our local institution; more than 10% of panel members represented external institutions to support generalizability. Our measure set was larger than planned, risking loss of focus on what contributes most to a high-performing oncology LHS. We believe, however, that addition of an eighth domain (diversity, equity, inclusion, and belonging) and the higher proportion of experience of care measures represent a strength of our measure set, prioritizing previously underappreciated features of a person-centered oncology LHS.

Measures are being deployed in a phased implementation process, with widespread deployment of some measures and small-scale testing of other measures. For example, at the health system level, we have incorporated collaboRATE (a measure of shared decision making) into the Consumer Assessment of Health Plans Study Clinician and Group Surveys and oncology postvisit experience surveys and have deployed employee surveys across the cancer center to capture Well-Being Index and Improvement Readiness. At a smaller scale, we have developed EHR capability to capture advance care planning and serious illness conversations and are pilot testing electronic collection of the Distress Thermometer and social determinants of health screening in several locations.

The resulting data and reporting infrastructure will support regular feedback of performance trends across the identified measure set using an audit and feedback approach (51) and will provide trends to target improvements in different domains. We are codesigning report prototypes, which highlight primary outcomes and allow users to drill down to access detailed data. A subset of Delphi panel participants is engaged in deploying measures and assessing performance of measures, with iterative adaptations over time.

Our person-centered oncology LHS value compass was codesigned by a diverse group of stakeholders. It will contribute to a deliberate approach to both measurement and reporting, feeding data back to multiple levels of the system and evaluating whether a balanced set of longitudinal measures can help drive practice-level and system-level improvements in health outcomes and experience, health-care quality, the well-being of health-care teams, and enhanced person-centered clinical and health services research and scholarship.

## Funding

This work was supported by the Gordon and Betty Moore Foundation (Grant #7485); the Robert Wood Johnson Foundation (Grant #75925); the National Cancer Institute at the National Institutes of Health (Grant P30 CA023108); and The Couch Fund at The Dartmouth Institute for Health Policy and Clinical Practice.

## Notes

**Role of the funder:** The funders had no role in the study design, collection, analysis, or interpretation of data; in the writing of the report; or in the decision to submit this work for publication.

**Disclosures:** Steven Leach is on the Medical Advisory Board of Nybo Therapeutics and is cofounder and Chair of the Scientific Advisory Board for Episteme Prognostics. Eugene Nelson holds stock in Quality Data Management, Inc. which provides information services on patient experience of care and data on quality of care.

**Author contributions:** Conceptualization: EN, KK, AT, MBF, AVC, AMK. Data curation: AVC and AMK. Formal analysis: AVC and AMK. Funding acquisition: SR, AT, SL, MBF, MMH, KK, EN. Investigation: AVC and AMK. Methodology: EN, AMK, AVC, KD, EM, AH, MBF, AT, KK. Project administration: AMK and MMH. Supervision: EN. Visualization: AVC. Writing—original draft: AVC, AMK, KK, AT, EN. Writing—review and editing: AVC, AMK, KK, KD, SL, MBF, EM, MMH, AH, EN, SR, AT. All authors read and approved the final manuscript.

**Acknowledgments:** The authors wish to acknowledge the contributions of The Promise Partnership Learning Health System data, measurement, and scholarship working group for their support in guiding this work and the members of the Promise Partnership Delphi Panel, including: Albert Mulley, Amber Barnato, Amelia Cullinan, Andrew Williams, Ann Bradley, Anna Tosteson, Anne Holmes, Anne Ireland, Brant Oliver, Brock Christensen, Carol Majewski, Carolyn Kerrigan, Catherine Reed, Cathy Morrow, Corey Siegel, Daniel Jantzen, David Finley, Elissa Malcolm, Elizabeth Bengtson, Elizabeth McGrath, Elizabeth Stedina, Ellen Flaherty, Elliott Fisher, Eric Henderson, Erick Lansigan, Evan Benjamin, Gabriel Brooks, Garret Wasp, George Blike, Ira Byock, Janet Haines, Jenn Alford-Teaster, Jenna Schiffelbein, Jennifer Snide, Joanna Leyenaar, Jocelyn Chertoff, Joga Ivatury, Johanna Beliveau, John Sweetenham, Judith Rees, Julia Dalphin, Julie Kim, Karen Clements, Kathryn Kirkland, Kenneth Meehan, Konstantin Dragnev, Kris Bowen, Lawrence Dacey, Linton Evans, Malavika Govindan, Marcus Thygeson, Martha Goodrich, Mary Chamberlin, MaryAnn Stump, Matthew Mackwood, Matthew Wilson, Meredith Sorensen, Michael Calderwood, Paul Barr, Polly Campion, Ralph Jean-Mary, Rian M. Hasson, Sai Cherala, Sally Kraft, Samuel Casella, Samuel Shields, Sandra Wong, Shoshana Hort, Stephanie Tomlin, Stephen Liu, Stephen LeBlanc, Steven Leach, Susan DiStasio, Susan Reeves, Virginia Reed, Wendy Wells, Whitney Hammond, and Yolanda Sanchez.

**Disclaimers:** Not applicable.

**Prior presentations:** Not applicable.

## Data Availability

The dataset is a summary of the data collected over the 3 rounds of the Delphi process. It is included in the Supplementary Materials (available online).

## Supplementary Material

pkac037_Supplementary_Data
