# Supplementary material for: Prioritizing Measures That Matter Within a Person-Centered Oncology Learning Health System
Source: JNCI Cancer Spectr. 2022 May 6;6(3):pkac037. doi: 10.1093/jncics/pkac037 (PMC9219163; doi:10.1093/jncics/pkac037)
Supplement: pkac037_Supplementary_Data [file pkac037_supplementary_data.pdf]

Supplementary Materials:

Supplementary Table 1: Round 1 Voting Results on Measurement Domains

Clinical health outcomes		n	Mean score (SD); Higher = better (1-9)	Proportion respondents scoring a 7-9	Number identifying subdomain as top 2 priority
	Clinical improvement	72	8.1 (1.2)	86.1	47
	Symptoms / signs of illness	72	7.7 (1.4)	80.6	27
	Disease activity	71	7.2 (1.5)	69	22
	Complications	72	7.7 (81.9)	81.9	16
	Mortality	72	7.9 (1.7)	83.1	15
	Morbidity	71	7.6 (1.5)	83.3	12
Health-related quality of life outcomes					
	General well-being / quality of life	72	8.5 (0.8)	97.2	61
	Physical function	72	8.2 (0.9)	98.6	32
	Emotional health	72	8.2 (0.9)	94.4	27
	Social / role functioning	72	7.7 (1.2)	84.7	22
	Spiritual health	72	6.8 (1.6)	58.3	1
Patient / family experience of care					
	Alignment of care with goals and preferences	71	8.5 (0.9)	95.8	42
	Access to care	72	8.1 (1.1)	88.9	26
	Patient / family overall satisfaction with care	71	7.8 (1.2)	85.9	24
	Continuity / transitions / coordination of care	71	7.9 (1.0)	85.9	13
	Relationship / communication with clinician	71	7.9 (1.1)	91.5	12
	Shared decision-making / decision quality	71	7.8 (0.9)	93	12
	Patient engagement / activation in care	69	7.8 (1.1)	89.9	11
	Confidence in self-management	71	7.2 (1.3)	73.2	6
Care team experience					
	Overall quality of work life	72	8.3 (1.0)	93.1	41
	Burnout	72	8.0 (1.2)	88.9	27
	Physical / psychological safety	71	8.0 (1.3)	83.1	22
	Professional fulfillment	72	7.9 (1.0)	93.1	21
	Joy in work	72	7.6 (1.3)	80.6	18
	Leadership qualities of supervisors	72	7.3 (1.6)	81.9	9
	Documentation burden	72	6.8 (1.4)	66.7	5
	Voluntary turnover among staff	72	7.1 (1.8)	75	2
Cost and utilization: Healthcare costs and utilization					
	Out-of-pocket expenditures	71	8.0 (1.3)	85.9	37
	End-of-life quality measures	70	7.9 (1.3)	88.6	29
	Total per capita healthcare expenditures from insurance	70	7.2 (1.7)	71.4	27
	Indirect societal costs	71	7.3 (1.4)	77.5	21
	ER utilization	72	7.5 (1.3)	79.2	16
	Days spent in the hospital	71	7.1 (1.4)	66.2	9
	Ambulatory visits	71	6.4 (1.6)	43.7	2
	Telehealth visits	71	6.1 (1.6)	40.8	0
Cost and utilization: Health system financial indicators					
	Operating margin	72	7.8 (1.4)	87.5	49
	Unreimbursed care (e.g., costs to healthcare system)	71	7.4 (1.5)	80.3	31

	Patient retention	69	6.7 (1.6)	60.9	30
	Clinical productivity	72	7.3 (1.6)	70.8	22
	Number of new patients	70	7.2 (1.3)	72.9	12
Research engagement & productivity					
	Grants	71	7.6 (1.5)	81.7	46
	Publications	71	7.5 (1.4)	76.1	34
	Mentoring relationships	71	7.3 (1.6)	77.5	23
	% staff salaries covered for research	69	6.8 (1.4)	59.4	20
	Developmental / pilot projects	71	7.1 (1.5)	71.8	13
	Presentations	70	6.5 (1.5)	54.3	5
	Editorial board / peer review participation	70	6.2 (1.4)	47.1	1
	Patent filings	69	5.5 (1.6)	26.1	1
Learning culture and community					
	Development of the learning health system community	71	8.0 (1.2)	88.7	34
	Learning with / from patients to inform care decisions & improve care delivery	71	8.0 (1.1)	91.5	33
	Using science and evidence base to inform care decisions	72	8.1 (1.1)	87.5	31
	Culture of continuous improvement and innovation	72	8.2 (1.0)	93.1	29
	Development of learners	72	7.5 (1.4)	75	19

Supplementary Table 2: Round 2 Voting Results

	n	Mean rating	SD	Proportion respondents scoring a 4 or 5	Number identifying subdomain as top 2 priority
Health-related quality of life - Patient-reported general well-being / quality of life, symptoms, or functional status (North)					
* Distress Thermometer & Problem List	70	3.8	0.9	65.2	34
* PROMIS 29	70	3.7	1.0	58	25
SF-12	71	3.6	1.0	57.1	25
* ESAS	70	3.6	0.8	55.1	16
PHQ-9	70	3.5	1.1	50.7	19
EQ5D	71	3.4	1.1	45.7	15
Clinical health outcomes - Mortality, safety, preventable harm (West)					
* Serious reportable safety events	71	4.2	0.8	84.5	44
* Hospital acquired infections	69	3.9	0.8	75.4	23
30-day mortality after high-risk cancer surgeries	69	3.6	0.9	60.9	16
Mean restricted survival time for patients receiving chemo for common metastatic cancers	70	3.6	1.0	58.6	20
* Population-level mortality: screen-detectable cancers	71	3.6	1.0	63.4	24
Modifiable 10-year risk of death	70	3.4	1.0	42.9	13
Clinical health outcomes - Clinician reported clinical improvement, disease activity, or symptoms / signs of illness (West)					
* Clinical status	72	4.1	1.0	71.8	51
Palliative Performance Scale	70	3.8	0.9	62.3	29
* ECOG Scale of Performance Status	71	3.7	0.9	64.3	30
Karnofsky Performance Status Scale	72	3.7	0.9	62	32
Health-related quality of life - Patient or caregiver reported burden, coping, and support (North)					
* Modified Caregiver Strain Index	71	3.9	0.9	73.2	44
Zarit Burden Interview	70	3.7	0.9	64.3	27
* Multidimensional Scale of Perceived Social Support	70	3.5	0.8	48.6	34
Brief COPE	69	3.4	0.9	43.5	26
Experience of care - Alignment of care with patient's goals and preferences (East)					
* Documentation of advance care plan	71	4.4	0.9	85.9	22
* collaboRATE	71	3.9	0.9	73.2	38
Shared decision-making index	70	3.6	1.0	57.1	34
Decision regret scale	70	3.2	0.9	37.1	10
Experience of care - Access to care, continuity of care, care integration (East)					
* New patients seen within 10 days, in cancer center	69	4.3	0.8	84.1	39
* Coordination of cancer care: Cancer Care Survey	68	4.2	0.9	79.4	43
Coordination of care for outpatients: CG-CAHPS	69	4.0	0.8	75.4	19
Continuity of care for hospitalized patients: HCAHPS	70	4.0	0.9	71.4	17
* Patients scheduled within 2 days of referral, in cancer center	70	3.9	1.0	68.6	21
Experience of care - Patient / family overall satisfaction with care (East)					
* consideRATE	67	3.9	0.9	74.6	41
* Multi-dimensional rating of cancer care experience	66	3.7	1.0	62.1	32
Overall rating of cancer care: Cancer Care Survey	67	3.6	0.9	52.2	27
Multi-dimensional rating: HCAHPS	65	3.4	1.0	43.1	10
Multi-dimensional rating: CG-CAHPS	67	3.4	0.9	41.8	10
Overall rating of outpatient provider: CG-CAHPS	65	3.3	0.9	35.4	6
Overall rating of hospital: HCAHPS	65	3.1	0.9	29.2	5
Team well-being / Joy in work (Northeast)					

* Well-being Index	70	4.3	0.7	90	49
* Voluntary turnover rate for staff with 1-5 years of experience	67	3.8	1.0	68.5	31
Press Ganey multi-dimensional measure of care team experience	68	3.7	1.0	58.8	26
Relational Coordination Survey	70	3.6	0.9	62.9	18
Safety culture subscale of Press-Ganey survey	70	3.5	1.0	50	14
SCORE work setting assessment	68	3.4	1.0	44.1	4
Costs, resource utilization, and health system financial indicators - Quality measures of care at the end-of-life (South)					
* Summary score for the end-of-life quality measures	68	4.3	0.9	80.9	44
* % died from cancer not enrolled in hospice	71	3.9	1.0	70.4	26
% admitted to the ICU in the last 30 days of life	71	3.9	1.0	70.4	21
% died from cancer enrolled in hospice for less than 3 days	70	3.8	1.0	70	19
% with 1+ emergency room visit in the last 30 days of life	71	3.7	0.9	60.6	9
% with receipt of chemotherapy in last 14 days of life	70	3.6	1.0	55.4	14
% with 1+ hospitalization in the last 30 days of life	71	3.5	0.9	49.3	5
Costs, resource utilization, and health system financial indicators - Costs of care (South)					
Total cost of care index	68	4.1	0.8	83.8	28
Total resource use index	69	4.0	0.8	73.9	28
* Out-of-pocket payments	70	3.9	0.9	70	32
Costs, resource utilization, and health system financial indicators - Financial toxicity (South)					
* % with financial toxicity referred to appropriate support or resources	70	4.4	0.7	92.9	42
* Avoidance/delay in accessing treatment due to cost worries	71	4.3	0.8	84.5	37
% screened for financial toxicity	72	4.2	0.8	86.1	36
COST-FACIT	71	4.0	0.8	71.8	25
Costs, resource utilization, and health system financial indicators - Financial health of the organization (South)					
Operating margin	69	4.2	0.9	82.6	52
Patient retention	70	4.0	0.8	77.1	33
Days cash on hand	67	3.9	1.0	76.1	35
Number of new patients	68	3.6	0.9	51.5	16
Research engagement & productivity (Southwest)					
* Multi-dimensional index of institutional commitment and support for research	70	4.3	0.9	85.7	53
* Multi-dimensional index of academic productivity	71	4.0	0.9	78.9	40
Overall satisfaction with institutional commitment and support for research	71	3.8	1.0	66.2	23
Return on investment of institutional support for research	70	3.7	0.9	68.6	14
Proportion of clinical trials that meet first enrollment within 60 days	70	3.3	1.0	44.3	8
Learning culture and community - Using science and evidence to inform care decisions (Southeast)					
* % of eligible patients screened for breast, cervical, or colorectal cancer, and/or tobacco use and cessation intervention	69	4.0	1.0	75	38
* Compliance with Commission on Cancer Quality of Care Measures	68	4.0	1.0	76.1	29
* Compliance with CMS' Core Set of Medical Oncology Quality Measures	68	3.8	0.9	68.7	32
Documentation of pain intensity and plan of care	66	3.6	1.1	56.9	15
Compliance with Joint Commission Elements of Performance relevant to cancer center	67	3.6	1.1	54.5	32
Learning culture and community - Culture of continuous improvement and innovation (Southeast)					
* % of staff completing 1+ professional career development opportunity	71	3.7	1.0	45.6	32
* Improvement Readiness Scale	71	3.6	1.1	58.6	29

* % of QI project teams that involve 1 patient or care partner	71	3.6	0.9	55.7	23
% of care team members who have completed a formal QI training program	69	3.4	1.0	62.9	20
MUSIQ	71	3.3	1.1	40	19
# of MOC Part IV QI projects and credits earned	71	2.9	1.0	25.7	4
Diversity, equity, inclusion, and belonging (Northwest)					
* Diversity, equity, and inclusion of workforce	71	4.3	0.9	88.6	53
* Inclusivity of patient population based on race, income, and education levels	70	4.0	1.0	72.5	30
Proportion of research portfolio focused on health equity, disparity research, etc.	70	3.8	1.0	65.2	24
Progress toward Healthcare Anchor Network goals	69	3.7	1.0	57.4	15
Charity care and other community benefit spending	70	3.5	0.9	55.1	13
Medicaid revenue as a share of patient revenue	70	3.2	1.0	39.1	1

* Measures advancing from Round 2 voting

Supplementary Table 3: Results of Round 3 Voting of Specific Measures

	Sub-domain & measure type *	n	Mean rank (Lower = better)	# identifying measure in top half of ranked items	% identifying measure in top half of ranked items
Health-related quality of life (North)					
PROMIS Global 10 [a]	A, Patient	70	2.6	48	69% ^
Distress Thermometer & Problem List	A, Patient	70	2.7	51	73% ^
ESAS	A, Patient	70	2.7	51	73%
Modified Caregiver Strain Index	B, Patient	70	3.7	33	47% ^
PROMIS Social Isolation	B, Patient	70	5.1	9	13%
PRO-CTCAE	A, Patient	70	5.1	11	16%
Single-item Social Isolation	B, Patient	70	6.1	6	9%
Clinical health outcomes (West)					
Clinical status (categories)	C, EHR	73	2.0	63	86%
ECOG Scale of Performance Status	C, EHR	73	2.9	53	73% ^
Survival (1, 3, 5 years by cancer type and stage of diagnosis)	D, Claims	73	3.7	41	56% ^
Serious reportable safety events	D, Admin	73	3.9	26	36%
Population-level mortality: screen-detectable cancers	D, Claims	73	4.2	22	30%
Healthcare acquired infections [b]	D, Admin	73	4.4	14	19%
Experience of care (East)					
collaboRATE	E-1, Patient	72	3.3	44	61% ^
Confidence to manage symptoms	F-1, Patient	72	3.3	46	64% ^
Coordination of cancer care: Cancer Care Survey	F-1, Patient	72	3.8	40	56% ^
consideRATE	G-1, Patient	72	3.5	26	36% $
Multi-dimensional rating of cancer care experience	G-1, Patient	72	4.3	26	36% ^
Overall rating of cancer care: Cancer Care Survey	G-1, Patient	72	4.9	19	26%
Likelihood to recommend cancer care center	G-1, Patient	72	5.0	15	21%
Access (appointment in 2 days, seen in 10 days) [c]	F-2, Admin	73	2.2	57	78% ^
Documentation of advance care plan	E-2, EHR	73	3.3	43	59% ^
Documentation of serious illness conversation	E-2, EHR	73	3.3	38	52% ^
Summary score for the end-of-life quality measures	H-2, Claims	73	3.6	36	49% ^
Financial toxicity (screening and action) [d]	I-2, EHR	73	3.6	35	48%
% died from cancer enrolled in hospice for less than 3 days	H-2, Claims	73	5.0	10	14%
Team well-being / Joy in work (Northeast)					
Well-being Index	Provider / Team	72	2.0	55	76% ^
Likelihood to recommend organization as a place to work	Provider / Team	72	2.3	37	51%
3R Questions: Respected, Resources, Recognized	Provider / Team	72	2.8	26	36%
Voluntary turnover rate for staff with 1-5 years experience	Admin	72	2.9	26	36% ^
Costs, resource utilization, and health system financial indicators (South)					
Avoidance/delay in accessing treatment due to cost worries	I, Patient	72	2.0	53	74% ^
Financial hardship (single item COST-FACIT) [e]	I, Patient	72	2.8	32	44% ^
Total cost of care index	J, Claims	72	3.2	24	33% ^
Out-of-pocket costs as fraction of total family income	J, Patient	72	3.4	14	19%
Total resource use index	J, Claims	72	3.7	21	29%
Scholarly engagement & productivity (Southwest)					

Multi-dimensional index of institutional commitment and support for research	Admin	72	1.7	60	83% ^
Multi-dimensional index of academic productivity	Admin	72	1.8	59	82% ^
% junior investigators meeting with mentor for a strategic research discussion quarterly	Admin	72	3.1	13	18%
% research portfolio focused on health equity or disparity research, health promotion or disease prevention research, social determinants of health, community health needs assessment, or community engaged research	Admin	72	3.4	12	17%
Learning culture and community (Southeast)					
Improvement Readiness Scale	K, Provider / Team	71	2.7	46	65% ^
% of QI project teams that involve 1+ patient or care partner	K, Admin	71	2.9	28	39%
% of staff completing 1+ professional career development opportunity	K, Admin	71	2.9	18	25%
Compliance with Commission on Cancer Quality of Care Measures	L, EHR	71	2.9	47	66% ^
Compliance with CMS' Core Set of Medical Oncology Quality Measures	L, EHR	71	2.9	43	61%
% of eligible patients screened for breast, cervical, or colorectal cancer, and/or tobacco use and cessation intervention	L, EHR, Registry	71	3.8	31	44%
Diversity, equity, inclusion, and belonging (Northwest)					
Diversity, equity, and inclusion of workforce	Admin	71	1.7	58	82% $
Inclusivity of patient population based on race, income, and education levels	EHR, Admin	71	2.8	30	42% ^
Proportion screened for social determinants of health	EHR	71	2.9	26	37% ^
Pay level of low-wage health workers	Admin	71	3.3	22	31%
% with a positive screen for any SDoH question	EHR	71	4.4	6	8%

Legend:

[a] Round 2 voting on PROMIS 29, modified to PROMIS 10 in round 3

[b] Round 2 voting on hospital-acquired conditions, modified to healthcare-acquired conditions

[c] Round 2 voting of two items (new patients seen within 10 days and patients scheduled within two days of referral); merged for round 3 voting

[c] Round 2 voting of two items (% with financial toxicity referred to appropriate support or resources; % screened for financial toxicity); merged for round 3 voting

[D] Round 2 voting on 12-item COST-FACIT, modified to single-item from COST-FACIT

^ Inclusion in final measure set

$ To be included within final measurement set, based on further refinement or testing by the healthcare system

* Sub-domains: A: Patient-reported general well-being / quality of life, symptoms, or functional status; B: Patient or caregiver reported burden, coping, and support; C: Clinician reported clinical improvement, disease activity, or symptoms / signs of illness; D: Mortality, safety, preventable harm; E: Alignment of care with patient's goals and preferences; F: Access to care, continuity of care, care integration; G: Patient / family overall satisfaction with care; H: Quality measures of care at the end of life; I: Financial toxicity; J: Costs of care; K: Culture of continuous improvement and innovation: L: Using science and evidence to inform care decisions; 1: Experience of care; 2: Care processes

Ballot 1

Promise Partnership Learning Health System

Value Measurement Set

The purpose of this modified Delphi process is to develop a small set of balanced measures that will be used to evaluate the effectiveness of the Promise Partnership Learning Health System at Dartmouth-Hitchcock Health, to be initially developed and tested for oncology patients.

The Promise Partnership is an initiative of Dartmouth-Hitchcock Health, the Norris Cotton Cancer Center, and The Dartmouth Institute for Health Policy & Clinical Practice to implement a learning health system that captures data generated during clinician and patient interactions and uses the data for multiple purposes. The learning health system is designed to impact a broad view of success and healthcare value across six distinct domains, including: (1) clinical and functional health outcomes; (2) patient and family care experience; (3) team well-being / joy in work; (4) cost, resource utilization, and financial indicators; (5) research engagement and productivity; and (6) learning culture and community.

Using the unique perspective you have been asked to bring to this work, please rate the following subdomains according to their importance to you.

Domain 1: Clinical and Functional Health Outcomes This domain includes (a) conventional clinical measures of health or disease; and (b) patient-reported measures of health-related quality of life.

A. Clinical Health Outcomes
How important is it to measure the following items from a clinical health outcomes perspective? If you feel there is a subdomain we missed, you may write it in and rate it.

		Extremely Unimportant			Neither Important nor Unimportant			Extremely Important	
	1	2	3	4	5	6	7	8	9
Clinical improvement									
Disease activity									
Symptoms / signs of illness									
Complications									
Morbidity *(e.g., the presence of a medical condition)*									
Mortality *(e.g., death)*									
Other									

Clinical and Functional Health Outcome Priority Areas

If you could choose only two subdomains from above, which would you choose?

Subdomain #1

▼ Clinical improvement ... Other

Subdomain #2

▼ Clinical improvement ... Other

B. Health-related Quality of Life

How important is it to measure the following items from a health-related quality of life perspective? If you feel there is a subdomain we missed, you may write it in and rate it.

	Extremely Unimportant				Neither Important nor Unimportant				Extremely Important
	1	2	3	4	5	6	7	8	9
Physical function									
Emotional health									
Social / role functioning									
Spiritual health									
General well-being / quality of life									
Other									

Health-related Quality of Life Priority Areas

If you could choose only two subdomains from above, which would you choose?

Subdomain #1

▼ Physical function ... Other

Subdomain #2

▼ Physical function ... Other

Domain 2: Patient and Family Care Experience This domain includes patients' and families' reports and perceptions on their care experiences (professional care received and self-care activities) including the patient's perceived health benefit, goal attainment, and preferences being met.

How important is it to measure the following items from a patient and family care experience perspective? If you feel there is a subdomain we missed, you may write it in and rate it.

	Extremely Unimportant				Neither Important nor Unimportant				Extremely Important
	1	2	3	4	5	6	7	8	9
Access to care									
Patient engagement / activation in care									
Relationship / communication with clinician									
Alignment of care with patient's goals and preferences									
Shared decision-making or decision quality									
Confidence in self-management of disease									
Continuity / transitions / coordination of care									
Patient / family overall satisfaction with care									
Other									

Patient and Family Care Experience Priority Areas

If you could choose only two subdomains from above, which would you choose?

Subdomain #1

▼ Access to care ... Other

Subdomain #2

▼ Access to care ... Other

Domain 3: Team Well-being / Joy in Work This domain includes clinicians' and care team members' reports and perceptions on their experiences working in the health system, including perceived physical and psychological safety, meaning and purpose in work, choice and control over time, experience and camaraderie with others at work, and perceptions that their work life is fair and equitable.

How important is it to measure the following items from a team well-being / joy in work perspective? If you feel there is a subdomain we missed, you may write it in and rate it.

	Extremely Unimportant	Neither Important nor Unimportant	Extremely Important
	1 2 3	4 5 6	7 8 9
Burnout			
Professional fulfillment			
Joy in work			
Physical / psychological safety in the workplace			
Leadership qualities of supervisors			
Voluntary turnover among staff			
Documentation burden			
Overall quality of worklife			
Other			

Team Well-being / Joy in Work Priority Areas

If you could choose only two subdomains above, which would you choose?
Subdomain #1

▼ Burnout ... Other

Subdomain #2

▼ Burnout ... Other

Domain 4: Cost, Resource Utilization, and Financial Indicators This domain includes direct healthcare expenditures paid by the patient out-of-pocket, expenditures by insurance on behalf of the patient, as well as utilization of healthcare services that drive expenditures.

A. Healthcare Costs and Utilization

How important is it to measure the following items from a healthcare costs and utilization perspective? If you feel there is a subdomain we missed, you may write it in and rate it.

	Extremely Unimportant			Neither Important nor Unimportant			Extremely Important		
	1	2	3	4	5	6	7	8	9
Total per capita healthcare expenditures from insurance									
Out-of-pocket expenditures									
Days spent in the hospital									
ER utilization									
Ambulatory visits									
Telehealth visits									
End-of-life quality measures *(e.g., chemotherapy last 14 days of life, ICU admission last 30 days of life, etc.)*									
Indirect societal costs *(e.g., days lost from work or school for patient or their care partners / caregivers)*									
Other									

Healthcare Costs and Utilization Priority Areas

If you could choose only two subdomains from above, which would you choose?

Subdomain #1

▼ Total per capita healthcare expenditures from insurance ... Other

Subdomain #2

▼ Total per capita healthcare expenditures from insurance ... Other

B. Healthcare System Financial Indicators

How important is it to measure the following items from a healthcare system financial indicator perspective? If you feel there is a subdomain we missed, you may write it in and rate it.

	Extremely Unimportant			Neither Important nor Unimportant			Extremely Important		
	1	2	3	4	5	6	7	8	9
Operating margin									
Unreimbursed care *(e.g., costs to the healthcare system)*									
Clinical productivity									
Number of new patients									
Patient retention									
Other									

Healthcare System Financial Indicator Priority Areas

If you could choose only two subdomains from above, which would you choose?

Subdomain #1

▼ Operating margin ... Other

Subdomain #2

▼ Operating margin ... Other

Domain 5: Research Engagement & Productivity This domain includes research engagement and productivity of clinicians, staff, and students within the healthcare system.

How important is it to measure the following items from a research engagement and productivity perspective? If you feel there is a subdomain we missed, you may write it in and rate it.

	Extremely Unimportant<br>1 2 3	Neither Important nor Unimportant<br>4 5 6	Extremely Important<br>7 8 9
Publications			
Grants (applications and funded proposals)			
Developmental / pilot projects (applications and initiated proposals)			
Presentations			
Editorial board / peer review participation			
Proportion of staff salaries covered / protected for research			
Mentoring relationships			
Patent filings			
Other			

Research Engagement and Productivity Priority Areas

If you could choose only two subdomains above, which would you choose?

Subdomain #1

▼ Publications ... Other

Subdomain #2

▼ Publications ... Other

Domain 6: Learning Culture and Community This domain includes the use of science to inform care; the development of a culture of continuous improvement; and the support of learning at all levels, including trainees and life-long learners.

Learning Culture and Community
How important is it to measure the following items from a learning culture and community perspective? If you feel there is a subdomain we missed, you may write it in and rate it.

	Extremely Unimportant 1 2 3 · 4 Neither Important nor Unimportant 5 · 6 7 Extremely Important 8 9
Development of learners *(e.g., curriculum, student ratings, learning behaviors, team skills)*	
Development of the learning health system community *(e.g., commitment to diversity and inclusion, support of community, transformation of work / team culture)*	
Using science and evidence base to inform care decisions	
Learning with and from patients to inform care decisions and improve care delivery	
Culture of continuous improvement and innovation	
Other	

Learning Culture and Community Priority Areas

If you could choose only two subdomains above, which would you choose?
Subdomain #1

▼ Development of learners *(e.g., curriculum, student ratings, learning behaviors, team skills)* ... Other

Subdomain #2

▼ Development of learners *(e.g., curriculum, student ratings, learning behaviors, team skills)* ... Other

You are now finished with the survey. You will be brought to a summary of your responses which you may print or save as a PDF for your reference on the July 30 discussion call. Thank you for your time.

Ballot 2

Promise Partnership Learning Health System Value Measurement Set
Ballot 2: Subdomains and Potential Measures

The purpose of this voting process is to develop a small set of balanced measures to evaluate the effectiveness of the Promise Partnership Learning Health System at Dartmouth-Hitchcock Health, to be initially developed and tested in oncology services patients.

In this second ballot, we ask you to re-consider each subdomain **using the unique perspective you have been asked to bring to this work.** Please rank each subdomain according to its importance.

You will then rate potential measures within each subdomain.

Where possible, measures are hyperlinked to supporting materials. Blue hyperlinks will open in a new browser window. Your survey progress will be saved if you leave the survey.

Measures marked with two asterisks (**) are routinely collected and monitored at a leadership level within Dartmouth-Hitchcock Health. Please evaluate their importance as you would the rest of the measures.

Domain 1: Clinical and Functional Health Outcomes This domain includes conventional clinician- or system-rated measures of health or disease, and patient-reported measures of health-related quality of life.

Drag the subdomains below to order them from most important (top) to least important (bottom).
______ Clinician-reported clinical improvement, disease-activity, or symptoms/signs of illness
______ Patient-reported general well-being / quality of life, symptoms, or functional status
______ Patient or caregiver-reported burden, coping, and support
______ Mortality, safety, or preventable harm

Domain 1: Clinical and Functional Health Outcomes
Please rate the importance of each potential measure, ranging from not at all important to extremely important.

Clinician-reported **clinical improvement, disease activity, or symptoms / signs of illness**

	Not at all important	Slightly important	Moderately important	Very important	Extremely important
ECOG Scale of Performance Status, a 0-5 score corresponding to level of self-care, participation in daily activities, and physical ability	○	○	○	○	○
Karnofsky Performance Status Scale, a 0-100 score corresponding to ability to do daily activities, need for assistance or care, and disease state	○	○	○	○	○
Palliative Performance Scale, a 0-100 score based on ambulation, activity level and evidence of disease, self-care, oral intake, and level of consciousness	○	○	○	○	○
Clinical status, as measured by no evidence of disease/remission, responding, stable disease, progressive disease, metastasis, local or regional recurrence/relapse	○	○	○	○	○

Select **up to two** measures that are most important to include.

- [ ] ECOG Scale of Performance Status
- [ ] Karnofsky Performance Status Scale
- [ ] Palliative Performance Scale
- [ ] Clinical status

Patient-reported **general well-being, quality of life, symptoms, or functional status**

	Not at all important	Slightly important	Moderately important	Very important	Extremely important
NCCN Distress Thermometer & Problem List, a 0-10 score of distress and a 40-item list of practical problems, family problems, emotional problems, spiritual or religious concerns, and physical problems	○	○	○	○	○
PHQ-9, a 9-item survey of depression symptoms	○	○	○	○	○
Edmonton Symptom Assessment Scale (ESAS-r), a 10-item survey of symptoms (e.g., pain, tiredness, appetite, well-being)	○	○	○	○	○
Promis 29, a 29-item survey of physical function, anxiety, depression, fatigue, sleep disturbance, participation in social roles, and pain intensity and interference	○	○	○	○	○
SF-12, a 12-item survey of general health, physical functioning, mental health, physical and emotional role functioning, social functioning, body pain, and vitality	○	○	○	○	○
EQ5D, a 5-item survey of mobility, self-care, usual activities, pain / discomfort, and anxiety / depression	○	○	○	○	○

Select **up to two** measures that are most important to include.

- [ ] NCCN Distress Thermometer & Problem List
- [ ] PHQ-9
- [ ] Edmonton Symptom Assessment Scale
- [ ] Promis 29
- [ ] SF-12
- [ ] EQ5D

Patient- or caregiver-reported **burden, coping, and support**

	Not at all important	Slightly important	Moderately important	Very important	Extremely important/
Zarit Burden Interview, a 12-item survey of caregiver burden addressing personal strain and role strain	○	○	○	○	○
Modified Caregiver Strain Index, a 13-item survey of caregiver burden addressing domains of financial, physical, psychological, social, and personal strain	○	○	○	○	○
Brief COPE, a 28-item survey of strategies for coping with stress (including 14 coping strategies, inclusive of spiritual and emotional support)	○	○	○	○	○
Multidimensional Scale of Perceived Social Support, a 12-item survey of social support	○	○	○	○	○

Select **up to two** measures that are most important to include.

- [ ] Zarit Burden Interview
- [ ] Modified Caregiver Strain Index
- [ ] Brief COPE
- [ ] Multidimensional Scale of Perceived Social Support

Measures for **mortality, safety events, or preventable harm**

	Not at all important	Slightly important	Moderately important	Very important	Extremely important
Number of serious reportable safety events **	○	○	○	○	○
Number of hospital-acquired infections **	○	○	○	○	○
30-day mortality after high-risk cancer surgeries	○	○	○	○	○
Population-level mortality for screen-detectable cancers	○	○	○	○	○
Mean survival time (restricted to a defined time period) for patients receiving chemotherapy for common metastatic cancers	○	○	○	○	○
Modifiable 10-year risk of death	○	○	○	○	○

Select **up to two** measures that are most important to include.

- ☐ Serious reportable safety events
- ☐ Hospital acquired infections
- ☐ 30-day mortality after high risk cancer surgeries
- ☐ Population level mortality for screen-detectable cancers
- ☐ Mean survival time for patients receiving chemotherapy for common metastatic cancers
- ☐ Modifiable 10-year risk of death

If we have missed a measure that is important to include within the **Clinical and Functional Health Outcomes** domain, please list it below and specify the subdomain it falls under.

____

____

If you have further feedback on the measures, including comments on criteria for inclusion or exclusion, please provide below.

____

____

Domain 2: Patient and Family Care Experience This domain includes patients' and families' reports and perceptions of their care experiences, including professional care received or self-care activities.

Drag the subdomains below to order them from most important (top) to least important (bottom).
______ Alignment of care with patient's goals and preferences
______ Patient / family overall satisfaction with care
______ Access to care, continuity of care, care integration

Domain 2: Patient and Family Care Experience Please rate the importance of each potential measure, ranging from not at all important to extremely important

Measures for **alignment of care with patients goals and preferences**

	Not at all important	Slightly important	Moderately important	Very important	Extremely important
collaboRATE, a 3-item survey of shared decision-making	○	○	○	○	○
Shared decision-making index, an 8-item supplement to the CAHPS Cancer Care Survey	○	○	○	○	○
Decision Regret Scale, a 5-item survey that measures distress or remorse after a healthcare decision	○	○	○	○	○
Documentation of advance care plan	○	○	○	○	○

Select **up to two** measures that are most important to include.

☐ collaboRATE

☐ Shared decision-making index

☐ Decision Regret Scale

☐ Documentation of advance care plan

Measures for **patient / family overall satisfaction with care**

	Not at all important	Slightly important	Moderately important	Very important	Extremely important
Overall rating of hospital, from HCAHPS survey (item #18) **	○	○	○	○	○
Overall rating of outpatient provider, from CG-CAHPS Survey (item #18) **	○	○	○	○	○

Overall rating of cancer care, from CAHPS Cancer Care Survey (item #42)	○	○	○	○	○
Multi-dimensional rating of the hospital experience, from HCAHPS Survey (29-items) **	○	○	○	○	○
Multi-dimensional rating of the outpatient primary or specialty care experience, from CG-CAHPS Survey (31-items) **	○	○	○	○	○
Multi-dimensional rating of the cancer care experience, tailored for surgery, drug therapy, or radiation therapy, from CAHPS Cancer Care Survey (56 items)	○	○	○	○	○
consideRATE, an 8-item measure of the care experience for people with a serious illness	○	○	○	○	○

Select **up to two** measures that are most important to include.

- ☐ Overall rating of hospital
- ☐ Overall rating of outpatient provider
- ☐ Overall rating of cancer care
- ☐ Multi-dimensional rating of hospital experience
- ☐ Multi-dimensional rating of outpatient primary or specialty care experience
- ☐ Multi-dimensional rating of the cancer care experience
- ☐ consideRATE

Measures for **access to care, continuity of care, care integration**

	Not at all important	Slightly important	Moderately important	Very important	Extremely important
Patients scheduled within 2 days of referral, within cancer center **	○	○	○	○	○
New patients seen within 10 days, within cancer center **	○	○	○	○	○
Continuity of care for hospitalized patients, including hospital staff providing written information about symptoms or health problems to watch for and	○	○	○	○	○

talking about availability of help after leaving the hospital, from the HCAHPS Survey (item #16-17)					
Coordination of care for outpatients, including provider knowing about patient's medical history, providing information about test results, and talking about medications being taken, from the CG-CAHPS Survey (items #13, 17, 20)	○	○	○	○	○
Coordination of cancer care, including provider knowing about patient's medical history, providing information about test results, and talking about medications being taken, from CAHPS Cancer Care Survey (items #21, 25, 27)	○	○	○	○	○

Select **up to two** measures that are most important to include.

- ☐ Patient referrals scheduled within 2 days
- ☐ New patients seen within 10 days
- ☐ Continuity of care for hospitalized patients
- ☐ Continuity of care for outpatients
- ☐ Continuity of cancer care

If we have missed a measure that is important to include within the **Patient and Family Care Experience** domain, please list it below and specify the subdomain it falls under.

____

____

If you have further feedback on the measures, including comments on criteria for inclusion or exclusion, please provide below.

____

____

Domain 3: Team well-being / joy in work This domain includes clinician's and care team member's reports of their experience working at the health system.
Please rate the importance of each potential measure, ranging from not at all important to extremely important.

Measures for **team well-being / joy in work**

	Not at all important	Slightly important	Moderately important	Very important	Extremely important
Well-being Index, a 9-item survey that measures dimensions of burnout, fatigue, quality of life, depression, anxiety/stress, meaning in work, and time for personal/family life **	○	○	○	○	○
Relational Coordination Survey, a 7-item survey, tailored to each clinical area, addressing team dynamics including frequency, timeliness, and accuracy of communication; collaboration in solving problems, understanding and respect for various roles of providers caring for patients; and sharing goals for patient care	○	○	○	○	○
Safety culture subscale of Press-Ganey survey **, including (a) prevention and reporting (8 items), (b) pride and reputation (4 items), and (c) resources and teamwork (7 items)	○	○	○	○	○
SCORE work setting assessment, a 50-item survey of safety, communication, operational reliability, and engagement	○	○	○	○	○
Press Ganey multi-dimensional measure of care team experience,** including 101 items and five subscales: 1) engagement, 2) power, 3) leader index, 4) resilience: (a) activation and (b) decompression; 5) safety culture: (a) prevention and reporting, (b) pride and reputation, and (c) resources and teamwork	○	○	○	○	○
Voluntary turnover rate for staff with 1-5 years experience (%) **	○	○	○	○	○

Select **up to two** measures that are most important to include.

- ☐ Well-being Index
- ☐ Relational coordination survey
- ☐ Safety culture subscale of Press Ganey survey
- ☐ SCORE work setting assessment
- ☐ Press Ganey multi-dimensional measure of care team experience
- ☐ Voluntary turnover rate for staff with 1-5 years experience

If we have missed a measure that is important to include within the **Team Well-being / Joy in Work** domain, please list it below.

____

____

If you have further feedback on the measures, including comments on criteria for inclusion or exclusion, please provide below.

____

____

Domain 4: Cost, resource utilization, and health system financial indicators This domain includes healthcare expenditures and use of healthcare services, and health system financial indicators

Drag the subdomains below to order them from most important (top) to least important (bottom).
______ Quality measures of care at the end-of-life
______ Financial toxicity
______ Costs of care
______ Financial health of the organization

Domain 4: Cost, resource utilization, and health system financial indicators Please rate the importance of each potential measure, ranging from not at all important to extremely important.

Quality measures of care at the end-of-life (based on claims data)

	Not at all important	Slightly important	Moderately important	Very important	Extremely important
Receipt of chemotherapy in last 14 days of life	○	○	○	○	○
Proportion of people who died from cancer not enrolled in hospice	○	○	○	○	○
Proportion of people who died from cancer enrolled in hospice for less than 3 days	○	○	○	○	○
Proportion of patients who died from cancer admitted to the ICU in the last 30 days of life	○	○	○	○	○
Proportion of patients with more than one emergency room visit in the last 30 days of life	○	○	○	○	○
Proportion of patients with more than one hospitalization in the last 30 days of life	○	○	○	○	○
Summary score for the end-of-life quality measures listed above	○	○	○	○	○

Select **up to two** measures that are most important to include.

- ☐ Receipt of chemotherapy in last 14 days of life
- ☐ Proportion of people who died from cancer not enrolled in hospice
- ☐ Proportion of people who died from cancer enrolled in hospice for less than 3 days
- ☐ Proportion of patients who died from cancer admitted to the ICU in the last 30 days of life
- ☐ Proportion of patients with more than one emergency room visit in the last 30 days of life
- ☐ Proportion of patients with more than one hospitalization in the last 30 days of life
- ☐ Summary score for the end-of-life quality measures listed above

Measures for **financial toxicity**

	Not at all important	Slightly important	Moderately important	Very important	Extremely important
Proportion of patients screened for financial toxicity	○	○	○	○	○
Proportion of patients with financial toxicity referred to appropriate support or resources	○	○	○	○	○
Comprehensive Score for Financial Toxicity (COST-FACIT), an 11-item survey of financial stressors	○	○	○	○	○
Avoidance or delay in accessing care or medications due to worry about cost, as measured by the National Health Interview Survey (9 items)	○	○	○	○	○

Select **up to two** measures that are most important to include.

- ☐ Proportion of patients screened for financial toxicity
- ☐ Proportion of patients with financial toxicity referred to appropriate support or resources
- ☐ COST-FACIT
- ☐ Cost-related care avoidance or delay in seeking care

Measures for **costs of care**

	Not at all important	Slightly important	Moderately important	Very important	Extremely important
Out-of-pocket payments: Direct payments made by individuals to health care providers at the time of service use over the last 30-days (including medications, co-payments, or other health care expenses)	○	○	○	○	○
Total cost of care index, a measure that reflects a mix of factors such as patient illness burden, service utilization and negotiated prices, including all costs associated with professional, facility inpatient and outpatient, pharmacy, lab, radiology, ancillary and behavioral health services	○	○	○	○	○
Total resource use index, a measure of the frequency and intensity of services used to manage a provider group’s patients, including all costs associated with professional, facility inpatient and outpatient, pharmacy, lab, radiology, ancillary and behavioral health services	○	○	○	○	○

Select **up to one** measure that is most important to include.

- ☐ Out-of-pocket costs
- ☐ Total cost of care index
- ☐ Total resource use index

Measures for **financial health of the organization**

	Not at all important	Slightly important	Moderately important	Very important	Extremely important
Operating margin, as defined by the difference between total operating revenues and total operating expenses (excluding investment income) **	○	○	○	○	○
Days cash on hand, as defined by the amount of cash and investments in the D-HH Balance Sheet divided by one day's worth of cash operating expenses **	○	○	○	○	○
Number of new patients	○	○	○	○	○
Patient retention	○	○	○	○	○

Select up to **two** measures that are most important to include.

- ☐ Operating margin
- ☐ Days cash on hand
- ☐ Number of new patients
- ☐ Patient retention

If we have missed a measure that is important to include within the **Cost, Resource Utilization, and Health System Financial Indicators** domain, please list it below and specify the subdomain it falls under.

____

____

If you have further feedback on the measures, including comments on criteria for inclusion or exclusion, please provide below.

____

____

Domain 5: Research engagement and productivity This domain includes institutional commitment and support for research and academic productivity.
Please rate the importance of each potential measure, ranging from not at all important to extremely important.

Measures for **research engagement and productivity**

	Not at all important	Slightly important	Moderately important	Very important	Extremely important
Multi-dimensional index of institutional commitment and support for research, including amount of pilot funding through institutional mechanisms; mechanisms and money for protected research time; investment in research education and support for clinical trials and related infrastructure, successful applications for extramural funding, and biostatistics and informatics support	○	○	○	○	○
Overall satisfaction with institutional commitment and support for research	○	○	○	○	○
Return on investment of institutional support for research, including ratio of institutional investment relative to yield in grant funding	○	○	○	○	○
Multi-dimensional index of academic productivity, including publications, grants, number of investigator-initiated clinical trials underway, and work that led to change in practice at our institution and beyond (e.g., lung cancer screening program based on own team's research findings)	○	○	○	○	○
Proportion of clinical trials that meet first enrollment within 60 days **	○	○	○	○	○

Select **up to two** measures that are most important to include.

- ☐ Index of institutional commitment to research
- ☐ Scholar satisfaction with institutional commitment and support for research
- ☐ Return on investment of institutional support for research
- ☐ Index of academic productivity
- ☐ Proportion of clinical trials that meet first enrollment within 60 days

If we have missed a measure that is important to include within the **Research Engagement & Productivity** domain, please list it below.

____

____

If you have further feedback on the measures, including comments on criteria for inclusion or exclusion, please provide below.

____

____

Domain 6: Learning culture and community This domain assesses the learning culture and community within the Promise Partnership learning health system.

Drag the subdomains below to order them from most important (top) to least important (bottom).
______ Culture of continuous improvement and innovation
______ Using science and evidence to inform care decisions

Domain 6: Learning culture and community Please rate the importance of each potential measure, ranging from not at all important to extremely important.
Measures of **culture of continuous innovation and improvement**

	Not at all important	Slightly important	Moderately important	Very important	Extremely important
Proportion of staff completing at least one professional career development opportunity **	○	○	○	○	○
Proportion of care team members who have completed a formal quality improvement training program	○	○	○	○	○
Proportion of quality improvement project teams that involve at least one patient or care partner	○	○	○	○	○
Number of maintenance of certification (MOC) Part IV quality improvement projects and credits earned	○	○	○	○	○
Improvement Readiness, a 5-item survey assessing the ability of the learning environment to support quality improvement	○	○	○	○	○
Model for Understanding Success in Quality (MUSIQ), a 35-item survey assessing contextual factors that influence the success of a quality improvement project	○	○	○	○	○

Select **up to two** measures that are most important to include.

- ☐ Staff completing 1+ professional career development opportunity
- ☐ % care team members completed a formal quality improvement training program
- ☐ % quality improvement project teams that include 1+ patient or care partner
- ☐ # of maintenance of certification (MOC) Part IV quality improvement projects and credits earned
- ☐ Improvement Readiness survey
- ☐ Model for Understanding Success in Quality (MUSIQ) survey

Measures for **using science and evidence to inform care decisions**

	Not at all important	Slightly important	Moderately important	Very important	Extremely important
Compliance with Joint Commission Elements of Performance relevant to cancer center **	○	○	○	○	○
Compliance with CMS' Core Set of Medical Oncology Quality Measures (14-items)	○	○	○	○	○
Compliance with Commission on Cancer Quality of Care Measures (23-items)	○	○	○	○	○
Documentation of pain intensity and plan of care	○	○	○	○	○
Screening and prevention: Proportion of eligible patients screened for breast cancer, cervical cancer, colorectal cancer, and / or tobacco use and cessation intervention	○	○	○	○	○

Select **up to two** measures that are most important to include.

- ☐ Compliance with Joint Commission Elements of Performance relevant to cancer center
- ☐ Compliance with CMS' Core Set of Medical Oncology Quality Measures
- ☐ Compliance with Commission on Cancer Quality of Care Measures
- ☐ Documentation of pain intensity and plan of care
- ☐ Screening and prevention: Proportion of eligible patients screened for breast cancer, cervical cancer, and/or colorectal cancer

If we have missed a measure that is important to include within the **Learning Culture and Community** domain, please list it below and specify the subdomain it falls under.

____

____

If you have further feedback on the measures, including comments on criteria for inclusion or exclusion, please provide below.

____

____

Domain 7: Diversity, equity, and inclusion This domain addresses diversity, equity, and inclusion of our patients, workforce, and community within the healthcare system and Promise Partnership learning health system.
Please rate the importance of each potential measure, ranging from not at all important to extremely important.

Measures for **diversity, equity, and inclusion**

	Not at all important	Slightly important	Moderately important	Very important	Extremely important
Diversity, equity, and inclusion of leaders, workforce, and trainees with respect to representation, retention, recruitment, selection, promotion, development, pay and benefits, and employee engagement (measurement to be defined by expert consultants)	○	○	○	○	○
Inclusivity, as defined by the extent to which the healthcare system's patient population reflects the demographics of the community in which it is located, based on race, income, and education levels (see page 6, Inclusivity)	○	○	○	○	○
Charity care and other community benefit spending	○	○	○	○	○

Medicaid revenue as a share of patient revenue	○	○	○	○	○
Progress toward Healthcare Anchor Network goals, including domains associated with hiring, purchasing, investing, population health, and sustainability	○	○	○	○	○
Proportion of research portfolio focused on health equity or disparity research, health promotion or disease prevention research, social determinants of health, community health needs assessment, or community engaged research	○	○	○	○	○

Select **up to two** measures that are most important to include.

- ☐ Diversity, equity, and inclusion of workforce
- ☐ Inclusivity
- ☐ Charity care and other community benefit spending
- ☐ Medicaid revenue as a share of patient revenue
- ☐ Progress toward Healthcare Anchor Network goals
- ☐ Proportion of research portfolio focused on health equity or disparity research, health promotion or disease prevention research, social determinants of health, community health needs assessment, or community engaged research

If we have missed a measure that is important to include within the **Diversity, Equity, and Inclusion** domain, please list it below and specify the subdomain it falls under.

____

____

If you have further feedback on the measures, including comments on criteria for inclusion or exclusion, please provide below.

____

____

You have now answered all questions in the survey.

You may review your responses by using the left or backward arrow.

TO COMPLETE THE SURVEY, click the right or forward arrow. You will be brought to a summary of your responses, which you may print or save as a PDF for your reference on the August 27 discussion call.

Thank you for sharing your time and expertise. We realize this was a big investment of your time, and thank you for your commitment to helping us develop measures to guide the D-HH Promise Partnership Learning Health System. We look forward to the next round of conversations.

Ballot 3

Promise Partnership Learning Health System Value Measurement Set
Ballot 3: Potential Measures

The purpose of this voting process is to develop a small set of balanced measures to evaluate the effectiveness of the Promise Partnership Learning Health System at Dartmouth-Hitchcock Health, to be initially developed and tested in oncology services.

In this third and final ballot, we ask you to re-consider each candidate measure **using the unique perspective you have been asked to bring to this work.** Please rank each measure according to its importance to you.

Where possible, measures are hyperlinked to supporting materials. Blue hyperlinks will open in a new browser window. Your survey progress will be saved if you leave the survey.

Clinical Health Outcomes
This domain includes conventional clinician- or system-rated measures of health, disease, safety, or adverse events.

Drag the measures below to order them from most important (top) to least important (bottom).
______ Clinical status, as measured by no evidence of disease/remission, responding, stable disease, progressive disease, metastasis, local or regional recurrence/relapse
______ ECOG Scale of Performance Status, a 0-5 score corresponding to level of self-care, participation in daily activities, and physical ability
______ Number of healthcare-associated infections
______ Number of serious reportable safety events
______ Population-level mortality for screen-detectable cancers
______ Survival (1, 3, 5 years by cancer type and stage of diagnosis)

If you have further feedback on the measures, including comments on criteria for inclusion or exclusion, or missing measures, please provide below.

____

Functional Health & Quality of Life Outcomes
This domain includes patient- or caregiver-reported measures of functioning or health-related quality of life.

Drag the measures below to order them from most important (top) to least important (bottom).
______ Distress Thermometer & Problem List, a 0-10 score of distress and a 40-item list of practical problems, family problems, emotional problems, spiritual or religious concerns, and physical problems
______ Edmonton Symptom Assessment Scale (ESAS-r), a 10-item survey of symptoms (e.g., pain, tiredness, appetite, well-being)
______ Modified Caregiver Strain Index, a 13-item survey of caregiver burden addressing domains of financial, physical, psychological, social, and personal strain
______ PRO-CTCAE (Patient-Reported Outcomes version of the Common Terminology Criteria for Adverse Events), a library of 124 items representing 78 symptoms (subsets of questions may be selected)
______ PROMIS Global-10, a 10-item survey assessing overall physical health, mental health, social health, pain, fatigue, and quality of life
______ PROMIS Social Isolation survey, a 4-item survey of feelings of social isolation
______ Social isolation single-item screening question, "How often do you feel isolated from others?"

If you have further feedback on the measures, including comments on criteria for inclusion or exclusion, or missing measures, please provide below.

____

Patient and Family Care Experience
This domain includes both (a) patients' and families' reports and perceptions of their care experiences, including professional care received or self-care activities, and (b) system ratings of care processes that impact the care experience.

Patient Perceptions
Drag the measures below to order them from most important (top) to least important (bottom).
______ collaboRATE, a 3-item survey of shared decision-making
______ Confidence to manage symptoms, a 4-item PROMIS survey of confidence or self-efficacy to manage symptoms
______ consideRATE, an 8-item measure of the care experience for people with a serious illness
______ Coordination of cancer care among doctors and other care providers, a 1-item rating from the Outpatient Oncology Survey (see Overall Assessment domain, question #1)
______ Likelihood to recommend cancer care center to others, a 1-item rating from the Outpatient Oncology Survey (see Overall Assessment domain, question #3)
______ Multidimensional rating of the cancer care experience, from the Outpatient Oncology Survey, including domains of scheduling, registration, facility, radiation therapy, chemotherapy/infusion, tests, oncologist, nurses, personal issues, and overall assessment
______ Overall rating of cancer care, a 1-item rating from the Outpatient Oncology Survey (see Overall Assessment domain, question #2)

If you have further feedback on the measures, including comments on criteria for inclusion or exclusion, or missing measures, please provide below.

____

Care Processes
Drag the measures below to order them from most important (top) to least important (bottom).
______ Proportion of people who are given a scheduled appointment within 2 days of referral and are seen by the care team within 10 days of referral, within cancer center
______ Proportion of patients not screened for financial toxicity, or screened and identified but not referred to appropriate support or resources
______ Proportion of people with documentation of advance care plan in electronic medical record
______ Proportion of people with stage 3 or 4 cancer or metastatic cancer with a serious illness conversation documented within the medical record
______ Proportion of people who died from cancer enrolled in hospice for less than 3 days
______ Summary of end-of-life quality measures: receipt of chemotherapy in last 14 days of life, died from cancer not enrolled in hospice, enrolled in hospice for less than 3 days, admitted to the ICU in the last 30 days of life, 1+ emergency room visit in the last 30 days of life, 1+ hospitalization in the last 30 days of life

If you have further feedback on the measures, including comments on criteria for inclusion or exclusion, or missing measures, please provide below.

____

Cost and Resource Utilization

This domain includes healthcare expenditures and use of healthcare services. *Previously this domain included financial health of the organization, with prioritization of operating margin and days cash on hand. These measures have been removed from the Promise Partnership Learning Health System ballot, however they remain part of Dartmouth Hitchcock Health's scorecard.*

Drag the measures below to order them from most important (top) to least important (bottom).

______ Avoidance or delay in accessing care or medications due to worry about cost, as measured by the National Health Interview Survey (9 items)

______ COST-FACIT: "My illness has been a financial hardship to my family and me", a 1-item measure from the Comprehensive Score for Financial Toxicity, see question #12

______ Out-of-pocket costs as a fraction of total family income: Direct payments made by individuals to health care providers at the time of service use over the last 30-days (including deductibles, co-insurance, and co-payments for covered services, plus all costs for services that aren't covered)

______ Total cost of care index, reflects a mix of complicated factors such as patient illness burden, service utilization and negotiated prices. Total Cost Index (TCI) is a measure of a primary care provider's risk-adjusted cost effectiveness at managing the population they care for. TCI includes all costs associated with treating members including professional, facility inpatient and outpatient, pharmacy, lab, radiology, ancillary and behavioral health services

______ Total resource use index, a risk-adjusted measure of the frequency and intensity of services utilized to manage a provider group's patients. Resource use includes all resources associated with treating members including professional, facility inpatient and outpatient, pharmacy, lab, radiology, ancillary and behavioral health services

If you have further feedback on the measures, including comments on criteria for inclusion or exclusion, or missing measures, please provide below.

____

Team well-being / Joy in Work

This domain includes clinician's and care team member's reports of their experience working within the healthcare system.

Drag the measures below to order them from most important (top) to least important (bottom).

______ Likelihood to recommend this organization as a good place to work, from the Press Ganey multidimensional measure of care team experience (see last question in the Engagement Indicator section)

______ 3R Questions: Respected, Resources, Recognized

______ Voluntary turnover rate for staff with 1-5 years experience (%)

______ Well-being Index, a 9-item survey that measures dimensions of burnout, fatigue, quality of life, depression, anxiety/stress, meaning in work, and time for personal/family life

If you have further feedback on the measures, including comments on criteria for inclusion or exclusion, or missing measures, please provide below.

____

Diversity, equity, and inclusion

This domain addresses diversity, equity, and inclusion of our patients, workforce, and community within the healthcare system and Promise Partnership learning health system.

Drag the measures below to order them from most important (top) to least important (bottom).

______ Diversity, equity, and inclusion of leaders, workforce, and trainees with respect to representation, retention, recruitment, selection, promotion, development, pay and benefits, and employee engagement (measurement to be defined by expert consultants)

______ Inclusivity, as defined by the extent to which the healthcare system's patient population reflects the demographics of the community in which it is located, based on race, income, and education levels (See page 6, Inclusivity)

______ Pay level of low-wage health workers (e.g., patient care assistants, home health or person care aides, food service workers, facility cleaning or maintenance)

______ Proportion of patients screened for social determinants of health

______ Proportion of patients with a positive screen for any social determinants of health question

If you have further feedback on the measures, including comments on criteria for inclusion or exclusion, or missing measures, please provide below.

____

Learning culture and community

This domain assesses the learning culture and community of practice within the Promise Partnership learning health system, including using science and evidence to inform care decisions, and the culture of continuous improvement and innovation.

Drag the measures below to order them from most important (top) to least important (bottom).

______ Compliance with CMS' Core Set of Medical Oncology Quality Measures (14-items)

______ Compliance with Commission on Cancer Quality of Care Measures (23-items)

______ Improvement Readiness, a 5-item survey assessing the ability of the learning environment to support quality improvement

______ Proportion of eligible patients screened for breast cancer, cervical cancer, colorectal cancer, and / or tobacco use and cessation intervention

______ Proportion of quality improvement project teams that involve at least one patient, family member, or care partner

______ Proportion of staff completing at least one professional career development opportunity

If you have further feedback on the measures, including comments on criteria for inclusion or exclusion, or missing measures, please provide below.

____

Research Engagement and Productivity
This domain includes institutional commitment and support for research and academic productivity.

Drag the measures below to order them from most important (top) to least important (bottom).
______ Index of academic productivity, including a locally-created index of publications, grants, number of investigator-initiated clinical trials underway, and work that led to change in practice at our institution and beyond (e.g., lung cancer screening program based on own team's research findings)
______ Institutional commitment and support for research, including a locally-created index of the amount of pilot funding through institutional mechanisms; mechanisms and money for protected research time; investment in research education and support for clinical trials and related infrastructure, successful applications for extramural funding, and biostatistics and informatics support
______ Proportion of junior investigators meeting with identified mentor for a strategic research discussion at least quarterly.
______ Proportion of research portfolio focused on health equity or disparity research, health promotion or disease prevention research, social determinants of health, community health needs assessment, or community engaged research

If you have further feedback on the measures, including comments on criteria for inclusion or exclusion, or missing measures, please provide below.

____

You have now answered all questions in the survey.
You may review your responses by using the left or backward arrow.
TO COMPLETE THE SURVEY, click the right or forward arrow. You will be brought to a summary of your responses, which you may print or save as a PDF for your reference on the September 17 discussion call.

Thank you for sharing your time and expertise. We realize this was a big investment of your time, and thank you for your commitment to helping us develop measures to guide the D-HH Promise Partnership Learning Health System. We look forward to the final round of conversations.
